# Mapping malaria by combining parasite genomic and epidemiologic data

**DOI:** 10.1186/s12916-018-1181-9

**Published:** 2018-10-18

**Authors:** Amy Wesolowski, Aimee R Taylor, Hsiao-Han Chang, Robert Verity, Sofonias Tessema, Jeffrey A Bailey, T Alex Perkins, Daniel E Neafsey, Bryan Greenhouse, Caroline O Buckee

**Affiliations:** 1 Department of Epidemiology, Johns Hopkins Bloomberg School of Public Health, Baltimore, MD USA; 2 Department of Epidemiology, Harvard TH Chan School of Public Health, Boston, MA USA; 3 Center for Communicable Disease Dynamics, Harvard TH Chan School of Public Health, Boston, MA USA; 4 Infectious Disease and Microbiome Program, Broad Institute, Cambridge, MA USA; 5 Medical Research Council Centre for Global Infectious Disease Analysis, Department of Infectious Disease Epidemiology, Imperial College, London, UK; 6 Department of Medicine, University of California – San Francisco, San Francisco, CA USA; 7 Program in Bioinformatics and Integrative Biology, University of Massachusetts, Worcester, MA USA; 8 Division of Transfusion Medicine, Department of Medicine, University of Massachusetts, Worcester, MA USA; 9 Department of Biological Sciences and Eck Institute for Global Health, University of Notre Dame, Notre Dame, IN USA; 10 Department of Immunology and Infectious Diseases, Harvard TH Chan School of Public Health, Boston, MA USA; 11 Chan Zuckerberg Biohub, San Francisco, CA 94158 USA

**Keywords:** Malaria, Parasite genomics, Spatial modeling, *Plasmodium falciparum*

## Abstract

**Background:**

Recent global progress in scaling up malaria control interventions has revived the goal of complete elimination in many countries. Decreasing transmission intensity generally leads to increasingly patchy spatial patterns of malaria transmission in elimination settings, with control programs having to accurately identify remaining foci in order to efficiently target interventions.

**Findings:**

The role of connectivity between different pockets of local transmission is of increasing importance as programs near elimination since humans are able to transfer parasites beyond the limits of mosquito dispersal, thus re-introducing parasites to previously malaria-free regions. Here, we discuss recent advances in the quantification of spatial epidemiology of malaria, particularly *Plasmodium falciparum*, in the context of transmission reduction interventions. Further, we highlight the challenges and promising directions for the development of integrated mapping, modeling, and genomic approaches that leverage disparate datasets to measure both connectivity and transmission.

**Conclusion:**

A more comprehensive understanding of the spatial transmission of malaria can be gained using a combination of parasite genetics and epidemiological modeling and mapping. However, additional molecular and quantitative methods are necessary to answer these public health-related questions.

## Background

### The spatial dimensions of malaria control and elimination strategies

**Fig. 1:**
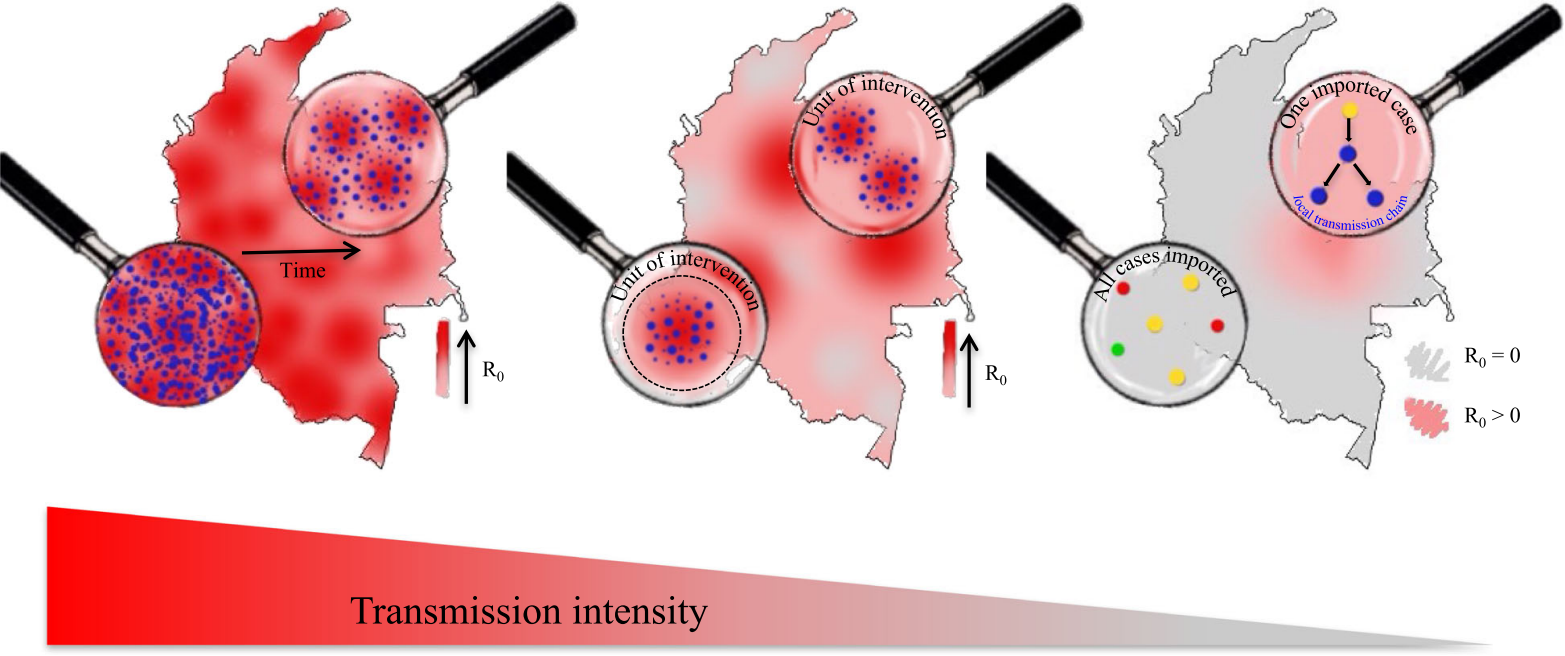
Actionable insight from genetic epidemiological studies of malaria across a range of transmission settings. This schematic depicts actionable insight that can be obtained from genetic epidemiological studies of malaria across a range of transmission settings, from high transmission (red) on the left to low transmission (gray) on the right. Here, both imported (stars) and local (points) infections, which may originate from different parasite lineages (various colors), are shown. In high transmission settings, parasites mix panmictically, polyclonal infections are common, and the goal is to evaluate the effectiveness of ongoing interventions. Genetic correlates of declining transmission (e.g., diversity) can provide sensitive indicators of the impact of an intervention. At intermediate transmission, parasites may cluster into interconnected populations. The goal is to delineate regions into units for targeted intervention and to identify the sources that seed transmission for maximally efficient resource allocation. In this setting, models incorporating human mobility and genetic measures of parasite relatedness can provide directional estimates of connectivity between parasite populations. At very low transmission, most infections are imported. The goal is to identify origins of imported parasites, quantify any onward transmission and, if onward transmission exists, the average length of local transmission chains. Models incorporating detailed case data, including genetic data and travel history, can reconstruct transmission chains to infer who acquires infection from who and how

Assessing variation in spatial and temporal patterns of infection or in the distribution of a particular pathogen phenotype, such as drug resistance, is an important prerequisite for any infectious disease control effort. For malaria, these considerations are critical across the range of transmission settings (Fig. 1). In pre-elimination settings (e.g., E-2020 countries, including Swaziland, Costa Rica, China, and South Africa [1]), surveillance programs must locate and track imported infections, conduct contact tracing, and ensure that onward transmission resulting from importation events are rapidly extinguished. For countries with intermediate transmission (e.g., Bangladesh, Namibia, and Thailand), control programs must identify the transmission foci contributing to infections in the rest of the country and locate importation hotspots since these will require approaches focused on transmission reduction like vector control. Even in high transmission settings (e.g., Uganda, Nigeria, Democratic Republic of Congo, and Myanmar), which have traditionally focused on monitoring clinical cases and scaling up control and treatment strategies across the country, the renewed interest in measuring transmission has also raised the possibility of more effective program evaluation to assess the impact of interventions on transmission in different regions. Of particular importance in moderate to high transmission settings is the coordination between different regions when human mobility between them is frequent.

### Model of malaria spatial epidemiology

A variety of modeling approaches has been used to describe the spatial dynamics of malaria [2] and to effectively allocate resources. Geostatistical modeling approaches have been used to generate maps of epidemiological variables such as parasite prevalence [3] and intervention impact [4]. These maps derive from methods that interpolate across spatially idiosyncratic data sources, providing a spatially smoothed estimate of epidemiological metrics relevant for targeting of interventions. Nevertheless, certain important aspects of malaria epidemiology cannot be captured by interpolation methods. First, statistical methods may fail to distinguish between areas where cases reflect local transmission intensity versus regions with frequently imported infections; therefore, different assumptions about connectivity can lead to varying conclusions with regard to the capacity for local transmission and need for vector control [5]. Second, thinking beyond all but the most local scales, there is a myriad of ways to coordinate control efforts across different areas, for example, by grouping locations that naturally cluster together as larger units of transmission [6, 7]. Combined with transmission models that consider numerous non-linear feedbacks between control and transmission [8, 9] and are capable of accounting for location-specific intervention packages and their impacts [10, 11], these approaches could, theoretically, suggest an optimal elimination strategy. In practice, there are shortcomings in both the currently available data and models.

Quantifying connectivity is one of the most important aspects of characterizing the spatial dynamics of malaria, yet it can be one of the most vexing. Call data records routinely collected by mobile phone operators, as well as other novel data sources on human travel, have offered hope in recent years [5, 7, 12]. These data are not without their challenges, however, including variable cell tower densities, mobile phone market fragmentation, and possible disconnects between who is making calls and who is transmitting parasites [13]. Traditional travel survey data may be more directly related to known symptomatic individuals; however, these data are often limited in scope and accuracy [14]. Understanding which travel patterns are epidemiologically relevant further requires an understanding of vector distribution, identity, and abundance. The complex relationship between these ecological parameters of transmission and the epidemiology of disease, along with the lack of robust parasite strain markers, make it difficult to accurately identify the geographical source of particular infections, in turn hindering efforts to map the routes of parasite importation at the population level. Ultimately, models are necessary to appropriately combine information about human mobility with a variety of epidemiological data to arrive at an estimate of how parasite movement arises on different spatial scales. Indeed, recent work using mathematical models based on epidemiological data in Senegal showed that genetic data collected in parallel can provide consistent and confirmatory signals of significant transmission reductions followed by signatures of a rebound [15]; similar approaches in a spatial context may well be useful in other settings.

Parasite genetic signals may offer some of the richest information about these otherwise elusive patterns of parasite movement and, although this approach is still in its early stages, researchers have begun to assess the utility of molecular surveillance as a routine tool for the optimization of control and elimination strategies. We propose that the marriage of parasite genetic data and models in a spatial context may offer unique insights into the epidemiology of malaria. Below, we discuss the techniques, challenges, and promising applications of molecular surveillance.

## Discussion

### Applications of parasite genetics to spatial epidemiology of malaria

Molecular tools may be most valuable when epidemiological information is scarce and/or mobility data is unavailable. Genomic surveillance and phylogenetic analyses that relate the geographic distribution of genetic signals within and between populations have enabled near real-time estimation of transmission chains for non-sexually recombining, rapidly evolving pathogens (e.g., Ebola, influenza) [16, 17]. This nascent field of pathogen phylogeography has provided key insights into the routes of pathogen introductions and spread, particularly for viral diseases. However, directly extending these methods to a pathogen such as *Plasmodium falciparum*—a sexually recombining eukaryotic parasite with a complex lifecycle—requires both molecular and analytic advancements that are still at the early stages of development. In particular, the malaria parasite *P*. *falciparum* undergoes obligate sexual recombination and is often characterized by multi-genotype infections and low-density chronic blood-stage infections that can last for months in asymptomatic individuals. More complex still are the many challenges associated with the second most abundant cause of malaria, *Plasmodium vivax* [18]. Unlike *P*. *falciparum* parasites, *P. vivax* parasites can survive for months or years as dormant hypnozoites in the liver, where they are undetectable, and can relapse and cause blood-stage infection at any time. Since genetically diverse hypnozoites can build up in the liver, relapses lead to an even greater abundance of multi-genotype blood-stage infections and thus more frequent recombination between genetically diverse parasites. Moreover, in regions of ongoing transmission, relapses cannot be definitely distinguished from reinfections due to new mosquito bites, further complicating efforts to spatially track *P*. *vivax* infection. These complexities mean that standard population genetic or phylogenetic approaches do not effectively resolve relationships between malaria parasite lineages [19]. Therefore, new tools are needed for the effective molecular surveillance of both parasite species.

Most national control programs are interested in spatial scales that are operationally relevant, namely within a given country or between countries if they are connected by migration. Population differentiation on international and continental geographic scales can be identified using principal component analysis, phylogenetic analysis, and the fixation index (*F*_*ST*_) [20–24], yet these methods are not powered to detect finer-scale differentiation. This is because (1) recombination violates the assumptions underpinning classic phylogenetic analyses [25], and (2) principal component analysis based on a pairwise distance matrix and *F*_*ST*_ is influenced by drivers of genetic variation that act on a long time scale (i.e., the coalescent time of parasites) such that if migration occurs multiple times during this time frame, there will be little or no signal of differentiation among populations [26, 27]. In contrast, methods that exploit the signal left by recombination (rather than treating it as a nuisance factor) may have the power to detect geographic differentiation on spatial scales relevant for malaria control programs.

Recombination occurs in the mosquito midgut when gametes (derived from gametocytes) come together to form a zygote. If the gametes are genetically distinct, recombination will lead to the production of different, but highly related, sporozoites (and thus onward infections). These highly related parasites would tend to have genomes with a high degree of identity. Perhaps the simplest measure of this genetic similarity is “identity by state” (IBS), which is defined as the proportion of identical sites between two genomes and is a simple correlate of genetic relatedness between parasites. However, IBS makes no distinction between sites that are identical by chance and those that are identical due to recent shared ancestry, making it sensitive to the allele frequency spectrum of the particular population under study. Analyses that are probabilistic (e.g., STRUCTURE [28]) provide better resolution, but ultimately linkage disequilibrium-based methods, such as identity by decent (IBD) inferred under a hidden Markov model [29, 30] and chromosome painting [31], provide greater power. These IBD methods harness the patterns of genetic linkage disequilibrium that are broken down by recombination and are therefore sensitive to recent migration events and useful at smaller geographic scales. Additionally, they take advantage of the signals present in long contiguous blocks of genomic identity, which can be detected given a sufficient density of informative markers. The exact density required is a topic of current research and depends on the level of relatedness, required precision, and the nature of the genetic markers in question (e.g., the number and frequency of possible alleles for each marker).

In low transmission settings, such as Senegal and Panama, STRUCTURE as well as IBS (which approximates IBD, albeit with bias and more noise), can often be used to cluster cases and infer transmission patterns within countries [32–34]. In intermediate transmission settings, such as coastal regions of Kenya and border regions of Thailand, where genetic diversity is higher, IBS, IBD, and relatedness based on chromosome painting have been shown to recover genetic structure over populations of parasites on local spatial scales [27, 35]. However, due to dependence on allele frequency spectra, IBS is not as easily comparable across datasets and, as mentioned above, can be overwhelmed by noise due to identity by chance. Moreover, all of these methods currently have limited support for polyclonal samples. In high transmission settings, the complexity of infection is very high, making it difficult to calculate genetic relatedness between parasites within polyclonal infections or to estimate allele frequencies across polyclonal infections since the complexity entangles the signal from the genetic markers belonging to the individual clones, the number of which is unknown. Methods to disentangle (i.e., phase) parasite genetic data within polyclonal infections are being developed [36], while THE REAL McCOIL [37] has been developed to simultaneously infer allele frequencies and complexity of infection, allowing downstream calculation of *F*_*ST*_. However, to fully characterize genetic structure at fine scales in high transmission settings, new methods that estimate IBD and other relatedness measures are needed to infer ancestry between polyclonal infections. Indeed, across all spatiotemporal scales and transmission intensities, we propose that rather than being defined by the transmission of discrete (clonal) parasite lineages, malaria epidemiology may be best characterized as the transmission of infection states, often comprised of an ensemble of parasites. Subsets of these ensembles are often transmitted together by a mosquito to another person, and therefore, the combination of alleles/parasites present in an infection state provides rich information about its origin(s) beyond the composition of individual parasites.

### Current sampling and sequencing strategies for genomic epidemiology of malaria

The use of genetic approaches described above will depend on the routine generation of parasite genetic data since any molecular surveillance system will improve with more data and must be tailored to the sampling framework and sequencing approach. To date, many studies attempting to obtain epidemiologic information from genomic data have taken advantage of existing samples rather than having sampling tailored to the questions and public health interventions of interest. This is understandable given that a number of these studies have been exploratory and that informed decisions regarding sampling require a priori empiric data on parasite population structure (unavailable in most places) and a predetermined analysis plan (difficult when analytical approaches are actively in development). A more direct/tailored study design should be possible as more parasite genomic data become available and analytical methods mature. However, in general, a greater sampling of infections will be required to answer fine-scale questions regarding transmission (e.g., whether infections are local versus imported, determining the length of transmission chains) than for larger-scale questions such as relative connectivity of parasite populations between distinct geographic regions. Now that sequencing can be performed from blood spots collected on filter papers or even rapid diagnostic tests, collecting samples from passively detected symptomatic cases at health facilities offers the most efficient means of collecting large numbers of infected cases, often with high parasite densities, thus making them easier to genotype. Nevertheless, while this may be sufficient to characterize the underlying parasite population in some settings and for some questions, in others, the capture of asymptomatic cases through active case detection may be essential to understand transmission epidemiology, e.g., to determine the contribution of the asymptomatic reservoir in sustaining local transmission.

The discriminatory power of the genotyping method will depend on the local epidemiology and transmission setting. The two most common genotyping approaches, namely relatively small SNP barcodes and panels of microsatellite markers [38], have been extensively used to monitor the changes in the diversity and structure of the parasite population. However, signals in these markers may not be sufficient to distinguish geographic origin and have limited resolution in certain transmission settings [37, 39, 40]. Increasing the number of loci and/or discrimination of each locus may be necessary to answer the questions relevant to elimination. Further, increasing discrimination by using multiallelic loci has particular advantages since these may provide more information content than biallelic loci [41]. This is particularly true in polyclonal infections, frequent even in areas close to elimination, because heterozygous genotypes of biallelic loci contain little information (all possible alleles are present), whereas detecting, for example, 3 out of 20 potential alleles in an infection, still allows informative comparisons between infecting strains. In addition, some genotypable multiallelic loci contain extremely high diversity, which can be combined in relatively small numbers to create high-resolution genotypes. Targeting specific regions of the genome for sequencing after amplification by PCR (amplicon sequencing) or other methods, such as molecular inversion probes [42], offers efficient approaches to genotyping multiallelic short-range haplotypes, SNPs, and/or microsatellites, providing a flexible platform for deeper and more consistent coverage of regions of interest at lower cost than whole genome sequencing. Amplicon sequencing may be of particular interest for genotyping minor strains in polyclonal infections and/or low-density samples, whereas molecular inversion probes may excel for more highly multiplexed marker assays where capturing low-density samples is not critical. Identifying a panel of optimally informative genetic markers to address a specific question remains a major challenge that must balance the cost, throughput, and discriminatory power. For example, at fine geographic scales, larger numbers of more closely spaced markers with representative coverage of the genome may be required in contrast to studies comparing distant parasite populations; the density at which infected individuals are sampled and the underlying diversity and genetic structure will also affect the number and type of loci required.

With proper consideration, a parsimonious set of genetic targets may be identified as useful to answer a number of general questions regarding malaria genomics. Nonetheless, the development of a marker toolbox and genotyping methods tailored to answering questions relevant for transmission at different spatial scales is an important goal. To this end, several ambitious sequencing studies have begun, and over 4000 *P*. *falciparum* genomes have been sequenced from different transmission settings around the globe (such as the Pf3K Project, https://www.malariagen.net/data/pf3k-pilot-data-release-3) [40, 43, 44]. These genetic data are all publicly available, providing a crucial framework to build upon when designing more local, sequence-based epidemiological studies that balance the trade-off between the number of genetic loci evaluated and the quality of the data (e.g., depth of sequence coverage) for each parasite sample. Genomic sequencing methods are evolving rapidly towards high-throughput and low-cost, deep sequencing approaches that can be performed on routinely collected patient samples, allowing for evaluation of even asymptomatic low-density infections, e.g., by selective enrichment of parasite DNA [45, 46]. These enrichment methods can exacerbate the non-uniformity of sequencing coverage variation across the parasite genome and can require specialized filters to remove erroneous heterozygous calls, yet they generally produce genotypes exhibiting very high concordance with those from samples sequenced via alternate means [46, 47]. Preferential amplification of dominant strains in a polyclonal infection (i.e., missing minority clones) and the inability to detect copy number variation have also been described as potential limitations of these selective enrichment methods [47]. Nevertheless, despite these limitations, these methods are enabling cost-effective whole genome sequences from routinely collected blood samples. Moving forward, we must ensure that rich metadata are made easily available in the context of genome sequences, so that links can be made to experimental, epidemiological, and ecological variables and models.

### Combining data layers to map malaria

**Fig. 2:**
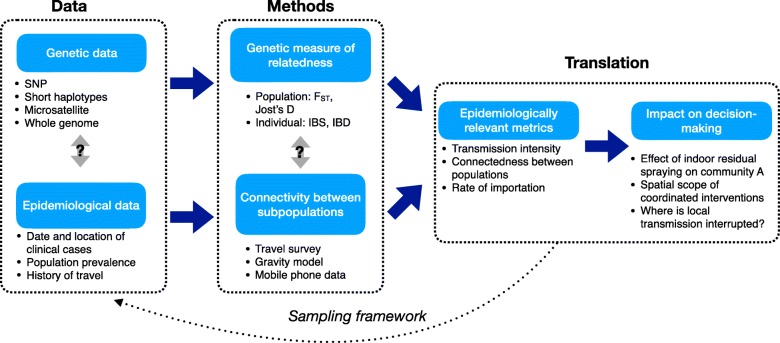
The analysis pipeline. Both genetic and epidemiological data can be collected and analyzed in order to understand the parasite flow (with example datasets and methods listed above). To identify how these two methods can be combined, directly related to policy-relevant questions, and translated to control measures will require the development of novel inference frameworks and the design of studies across a range of transmission settings

In concrete terms, we want to be able to clearly identify if two locations are epidemiologically linked. However, given the current methods available and in development, the complicated life cycle of the parasite, and the epidemiology of malaria, any single data source or method is unlikely to produce a complete picture of the spatial dynamics of malaria parasites. Figure 2 illustrates an analytical pipeline linking different spatially explicit datasets to methods and ultimately interventions, highlighting current uncertainties and the need to consider policy-relevant metrics when designing sampling frameworks. In particular, we believe that future development should focus on identifying how these different types of data can be combined and integrated to provide a more complete picture of connectivity and transmission dynamics. If we view this problem in terms of a simplified traditional medical statistic, malaria parasite data have a high false-negative rate (the analysis mostly underestimates relatedness between parasites), whereas connectivity data inferred from mobile phone data or other proxy measures of travel have a high false-positive rate (the analysis mostly overestimates the number of epidemiologically relevant connections). Ideally, joint inference methods that combine these data sources would help improve the type I (false-positivity rate) and type II (false-negativity rate) errors in each type of data.

## Conclusions

These new data streams therefore offer great potential, but understanding how to effectively combine them in ways that consider the biases and strengths of each data type will require significant research investment. Furthermore, making these methods relevant for implementation is a consideration that must be at the forefront of research efforts. For example, the ongoing availability of each data stream, the feasibility of implementing these analytical approaches in the context of national control programs as well as the capacity-building required to do so, will ultimately determine their impact. This means that tools must provide clearly communicated estimates of uncertainty and will need to be straightforward for their use in different contexts, easy to communicate, and generalizable.

## Abbreviations

*F*_*ST*_: Fixation index
IBD: Identical by descent
IBS: Identical by state
