# Correction to: Mapping malaria by combining parasite genomic and epidemiologic data

**DOI:** 10.1186/s12916-018-1232-2

**Published:** 2018-12-28

**Authors:** Amy Wesolowski, Aimee R. Taylor, Hsiao-Han Chang, Robert Verity, Sofonias Tessema, Jeffrey A. Bailey, T. Alex Perkins, Daniel E. Neafsey, Bryan Greenhouse, Caroline O. Buckee

**Affiliations:** 1 Department of Epidemiology, Johns Hopkins Bloomberg School of Public Health, Baltimore, MD USA; 2 Department of Epidemiology, Harvard TH Chan School of Public Health, Boston, MA USA; 3 Center for Communicable Disease Dynamics, Harvard TH Chan School of Public Health, Boston, MA USA; 4 Infectious Disease and Microbiome Program, Broad Institute, Cambridge, MA USA; 5 Department of Infectious Disease Epidemiology, Medical Research Council Centre for Global Infectious Disease Analysis, Imperial College, London, UK; 6 Department of Medicine, University of California–San Francisco, San Francisco, CA USA; 7 Program in Bioinformatics and Integrative Biology, University of Massachusetts, Worcester, MA USA; 8 Division of Transfusion Medicine, Department of Medicine, University of Massachusetts, Worcester, MA USA; 9 Department of Biological Sciences and Eck Institute for Global Health, University of Notre Dame, Notre Dame, IN USA; 10 Department of Immunology and Infectious Diseases, Harvard TH Chan School of Public Health, Boston, MA USA; 11 Chan Zuckerberg Biohub, San Francisco, CA 94158 USA

**Correction to: BMC Med**

**https://doi.org/10.1186/s12916-018-1181-9**

The original article [1] contained an error in the presentation of Figure 1; this error has now been rectified and Figure 1 is now presented correctly.
